# Supplementary material for: High Glucose-Induced Cardiomyocyte Damage Involves Interplay between Endothelin ET-1/ETA/ETB Receptor and mTOR Pathway
Source: Int J Mol Sci. 2022 Nov 10;23(22):13816. doi: 10.3390/ijms232213816 (PMC9699386; doi:10.3390/ijms232213816)
Supplement: Supplementary file 1 [file ijms-23-13816-s001.zip › ijms-1998385-supplementary.pdf]

Supplementary information

Supplementary table

Table S1. List of primer sequences (rat)

Name	Sequence (5' - 3')
F-ET_A-R	CGTCTTCTGCTTGGTTGTCA
R-ET_A-R	GCAACAGAGGCATGACTGAA
F-ET_B-R	CTGTGGGGATCACAGTGTTG
R-ET_B-R	TGCATGAAGGCTGTTTTCTG
F-ET-1	ACCACAGACCAAGGGAACAG
R-ET-1	GGTCTTGATGCTGTTGCTGA
F-GAPDH	AGACAGCCGCATCTTCTTGT
R-GAPDH	CTTGCCGTGGGTAGAGTCAT
F-mTOR	AGGGCAGCAACAGTGAAAG
R-mTOR	CGACAAGGAGATAGAACGGAAG
F-Raptor	CTCTCAACTGCCAACCATC
R-Raptor	AAAATCGTCCCAGCAAGTC
F-Rictor	ACACCATCCTTCCTCACTC
R-Rictor	AGGTCCTCGTTTCTTCATTTC
F-PGC1α	ATGTGTCGCCTTCTTGCTCT
R-PGC1	CGAGAAAAGGATCTCGAACG
F-TFAM	GGAAGAGCAAATGGCTGAAG
R-TFAM	CCCAATCCCAATGACAACTC

Supplementary figures

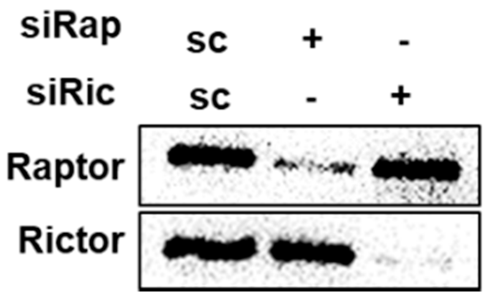

Figure S1. Silencing efficiency of siRNAs targeting raptor (siRap) and rictor (siRic)

The H9c2 cells were seeded in 10 cm culture dish in low glucose DMEM medium for overnight. Next, cells were transfected with siRNAs targeting either Raptor or Rictor (20 nM) or control siRNAs (sc) for 24 hours using Lipofectamine reagent. Thereafter, protein was harvested using lysis buffer described in methodology section. The western blotting was performed using primary antibodies specific for Raptor and Rictor.
